# Circulating tumor DNA predicts efficacy of a dual AKT/p70S6K inhibitor (LY2780301) plus paclitaxel in metastatic breast cancer: plasma analysis of the TAKTIC phase IB/II study

**DOI:** 10.1002/1878-0261.13188

**Published:** 2022-03-30

**Authors:** Renaud Sabatier, Cécile Vicier, Séverine Garnier, Arnaud Guille, Nadine Carbuccia, Nicolas Isambert, Florence Dalenc, Marie Robert, Christelle Levy, Jihane Pakradouni, José Adelaïde, Max Chaffanet, Patrick Sfumato, Emilie Mamessier, François Bertucci, Anthony Goncalves

**Affiliations:** ^1^ CRCM—Predictive Oncology Laboratory Institut Paoli‐Calmettes Inserm CNRS Aix‐Marseille Université France; ^2^ Institut Paoli‐Calmettes—Department of Medical Oncology CRCM Inserm CNRS Aix‐Marseille Université France; ^3^ Drug Development Department Centre Georges François Leclerc Dijon France; ^4^ Department of Medical Oncology Institut Claudius Regaud CRCT Inserm IUCT‐Oncopole Toulouse France; ^5^ Institut de Cancérologie de l'Ouest‐René Gauducheau Saint‐Herblain France; ^6^ Department of Medical Oncology Centre François Baclesse Caen France; ^7^ 56181 Depatment of Clinical Research and Innovation Institut Paoli‐Calmettes Marseille France

**Keywords:** AKT, breast cancer, circulating tumor DNA, copy number alterations, low‐coverage whole genome sequencing, survival

## Abstract

The phosphatidylinositol‐3‐kinase (PI3K)/AKT/mammalian target of rapamycin (mTOR) pathway is frequently activated in HER2‐negative breast cancer and may play a role in taxane resistance. The phase IB/II TAKTIC trial (NCT01980277) has shown that combining a dual AKT and p70 ribosomal protein S6 kinase (p70S6K) inhibitor (LY2780301) taken orally with weekly paclitaxel in HER2‐negative advanced breast cancer is feasible, with preliminary evidence of efficacy. We wanted to explore whether circulating tumor DNA (ctDNA) may be a surrogate marker of treatment efficacy in this setting. Serial plasma samples were collected and cell‐free DNA was sequenced using low‐coverage whole‐genome sequencing, and analysis was completed with droplet digital polymerase chain reaction (PCR) for some patients with driver mutations. Baseline tumor fraction (TF) and TF after 7 weeks on treatment were compared to progression‐free survival (PFS) and the overall response rate. We also explored circulating copy number alterations associated with treatment failure. Of the 51 patients enrolled in the TAKTIC trial, at least one plasma sample was available for 44 cases (96 timepoints). All patients with tumor *TP53*, *PI3KCA,* or *AKT1* mutations harbored at least one of these alterations in plasma. TF at inclusion was correlated with PFS (6m‐PFS was 92% for ctDNAneg patients *vs* 68% for ctDNApos cases; hazard ratio [HR] = 3.45, 95% confidence interval [CI] [1.34–8.90], *P* = 0.007). ctDNA status at week 7 was not correlated with prognosis. Even though most circulating copy number alterations were conserved at disease progression, some genomic regions of interest were altered in post‐progression samples. In conclusion, ctDNA detection at baseline was associated with shorter PFS in patients included in the TAKTIC trial. Plasma‐based copy number analysis may help to identify alterations involved in resistance to treatment.

## Graphical abstract

We used whole‐genome sequencing of circulating tumor DNA (ctDNA) to obtain copy number alteration profiles from the plasma of patients with metastatic breast cancer receiving a combination of paclitaxel and LY2780301 (dual AKT/p70S6H inhibitor). ctDNA detection at baseline predicted shorter progression‐free survival. Post‐progression plasma copy number alteration analysis may identify genomic alterations involved in resistance to treatment.

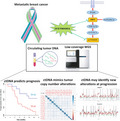

**Abbreviations**
CNA: copy number alterations
ctDNA: circulating tumor DNA
ddPCR: droplet digital polymerase chain reaction
ER: estrogen receptor
GISTIC: Genomic Identification of Significant Targets in Cancer
LC‐WGS: low‐coverage whole genome sequencing
MAF: mutant allele fraction
MBC: metastatic breast cancer
ORR: objective response rate
PFS: progression‐free survival
TF: tumor fraction
TNBC: triple‐negative breast cancer

## 1 Introduction

Metastatic breast cancer (MBC) is the second cause of death by cancer in women in Western countries [1]. Despite recent advances, treatment for MBC is still palliative and the 5‐year survival rate for MBC remains low (26%) [2]. The phosphatidylinositol‐3‐kinase (PI3K)/AKT/mammalian target of rapamycin (mTOR)‐pathway is frequently activated in HER2‐negative breast cancer [3]. It is associated with breast carcinogenesis, endocrine receptors positivity, survival, and resistance to endocrine therapies [4, 5]. Moreover, it may play a role in resistance to chemotherapy, including taxanes that are among the main cytotoxic drugs used for MBC [6].

To date, alpelisib is the only treatment targeting PI3K that has been described to improve MBC survival in combination with endocrine therapy [7]. Its efficacy is restricted to *PI3KCA* mutated tumors. Other compounds targeting this pathway are under development, including AKT inhibitors [8]. LY2780301, a dual inhibitor of p70 S6 kinase and AKT, has been shown to be safe as monotherapy [9], as well as in association with gemcitabine in patients with advanced solid tumors harboring PI3K/AKT/mTOR pathway alterations [10]. In the latter trial, the response rate was low, with only two patients (5% of the whole population) achieving a partial response. Nevertheless, the TAKTIC phase IB/II trial has recently reported a promising objective response rate (ORR) of 64% in combination with chemotherapy in HER2‐negative MBC [11]. Other AKT inhibitors have been explored to treat breast cancer. The ipatasertib‐paclitaxel combination failed to improve the pathological response rate after neoadjuvant therapy for early triple‐negative breast cancer (TNBC), but led to better survival in metastatic TNBC [12, 13]. Capivasertib did not improve progression‐free survival (PFS) nor in the overall population, neither in *PIK3CA*‐mutated tumors when combined with paclitaxel in HER2‐negative, estrogen receptor‐positive MBC [8]. However, it improves PFS in association with paclitaxel in metastatic TNBC and with fulvestrant in estrogen receptor (ER)‐positive tumors [14, 15]. In these trials, discrepant results have been observed concerning P3K/AKT/mTOR pathway alterations ability to predict AKT inhibitors efficacy [8, 14, 15].

Molecular alterations identification using circulating tumor DNA (ctDNA) can be used to monitor treatment efficacy. This has been proven for many tumor localizations such as breast, colon, or lung cancers. For breast cancer, it has been shown that ctDNA evolution rates are more sensitive to detect disease modifications than the currently used serum markers such as Ca15‐3 [16, 17]. Similar results had been published for lung [18] and colorectal cancers [19]. CtDNA is also routinely used as a predictive marker for some subpopulations such as *EGFR*‐mutated lung cancer for which EGFR inhibitors can be administered based on plasma analysis [20]. Concerning MBC, the US Food and Drug Administration approved both alpelisib and a companion diagnostic test (therascreen PIK3CA RGQ PCR Kit, Qiagen, Chatsworth, CA, USA) to detect *PIK3CA* mutations in tumor and/or plasma samples. Plasma analyses of the SOLAR1 trial indeed showed that alpelisib efficacy can be predicted by the *PIK3CA* mutation status in plasma [21].

The aim of this work was to assess the predictive value of ctDNA monitoring for patients receiving LY2780301‐paclitaxel for HER2‐negative MBC. Our primary objectives were to correlate prognosis with the ctDNA status at inclusion and to assess the predictive value of ctDNA after 7 weeks on treatment. Using low‐coverage whole‐genome sequencing at both baseline and progression, we also aimed to detect emerging alterations involved in resistance to treatment.

## 2 Materials and methods

### 2.1 Patients' selection and study design

All patients analyzed in this work were included in the TAKTIC‐IPC 2012‐008 study (N°EUDRACT 2013‐000585‐12, NCT01980277) and gave their consent for participating to ancillary studies. TAKTIC was a two‐step trial. The phase IB aimed to determine the recommended phase II dose (RP2D) of LY2780301 in combination with paclitaxel for patients with locally advanced or metastatic HER2‐negative breast cancer. Phase II focused on patients in the first‐line setting and aimed to estimate the best ORR of this combination in the overall population and in patients with activation of the PI3K/AKT/S6 pathway. Details of inclusion criteria, treatment regimen, and clinical results can be found elsewhere [11]. Details of tumor genomic analysis can also be found in the article related to the main clinical endpoints of the TAKTIC trial. This *post‐hoc* prespecified ancillary study was focused on ctDNA analysis of prospectively collected samples with annotated clinical data.

### 2.2 Plasma samples collection and cell‐free DNA extraction

Peripheral blood was collected in four 5 mL EDTA tubes at inclusion or cycle 1 day 1 (C1 D1), at C3 D1 (i.e., after 7 weeks of treatment), and at end of treatment (with a maximum of 18 months after treatment initiation). Plasma was separated within 2 h after venipuncture and stored at –80 °C. Cell‐free DNA (cfDNA) was isolated from plasma using the Maxwell (Promega, Madison, WI, USA) and Maxwell RSC circulating cell‐free DNA Plasma Kit (Promega) and quantified by the Qubit fluorometer (QuBit HS dsDNA kit; Thermo Fisher Scientific, Waltham, MA, USA), according to the manufacturers' instructions. Cell‐free DNA was then stored at –20 °C before further analyses.

### 2.3 Low‐coverage whole‐genome sequencing

We explored copy number abnormalities using low‐coverage whole‐genome sequencing analysis (LC‐WGS). Libraries were constructed using a commercially available Diagenode kit (MicroPlex Library Preparation Kit v2, Diagenode, Liège, Belgium) in accordance with the manufacturer's instructions. The cfDNA input was 5 ng per library preparation. The quality and quantity of each library were evaluated by the Agilent 2200 TapeStation System (Agilent HS D1000 Assay Kit; Agilent, Santa Clara, CA, USA) and the Qubit Fluorometer (Qubit dsDNA BR Assay Kit; Thermo Fisher Scientific), respectively. Each library was then reduced to four nanomolar before being pooled in equimolar amounts (12 libraries per mix). All library mixes were sequenced on a NextSeq500 Next Generation Sequencer (NGS) from Illumina (San Diego, CA, USA) with an average depth of coverage of 0.4×, generating readings of 2*75 base pairs (bp). We then determined the cfDNA fraction from tumor cells (TF, tumor fraction) for each timepoint. Reads were aligned with the human reference genome (hg19) using bwa software (v. 0.7.15‐r1140). Alignment was then processed to remove the duplicate sequences with the picard software (v. 2.9.2). A wig file containing the number of reads for regular intervals of 50 000 bp was generated with the readcounter software. Finally, TF evaluation was obtained using the ichorcna software (v. 0.3.2, Broad Institute, Cambridge, MA, USA). All LC‐WGS experiments were performed in simplicate.

For each sample, the genomic profile was established. To identify recurrent copy number alterations, we used the Genomic Identification of Significant Targets in Cancer (GISTIC) 2.0 algorithm [22], calculated by multiple random iterations, with an amplification/deletion threshold >0.1, confidence level 0.90, and a corrected threshold probability *q* < 0.25. We computed the percentage of concordance between baseline and progression for each significant region. Gained regions were consistent if gained in both samples (copy number ≥3), and lost regions were consistent if lost in both samples (copy number ≤1). Recurrent altered regions in progression samples were identified by exploring the number of discrepancies between baseline and progression, and *P*‐values were computed with a bootstrap procedure.

### 2.4 Single nucleotide mutations analyses

Cell‐free DNA was also assessed by digital droplet polymerase chain reaction (ddPCR) (Bio‐Rad QX200, Hercules, CA, USA) according to the manufacturer's instructions. Somatic tumor mutations had been explored using panel‐based sequencing on tumor samples collected at inclusion as previously described [11, 23]. We selected cases with mutations identified in *PI3KCA* hotspots (p.R88Q, p.E542K, p.E545K, p.H1047L, and p.H1047R), *AKT1* hotspot (p.E17K), and *TP53*. Selected mutations were used for ctDNA monitoring using ddPCR. Each timepoint was analyzed at least in duplicate. Reactions were assembled with 10 µL Bio‐Rad ddPCR supermix for probes, 2 µL mutation assay (1 µL each for the mutated and wildtype sequence) and 9 µL DNA and water volume. The reaction was partitioned into a maximum of 20 000 droplets on a Bio‐Rad AutoDG. The generated droplets were transferred to a 96‐well PCR plate. After thermal‐cycling (95 °C 10 min [1 cycle], 94 °C 30 s [ramp rate 2 °C·s^–1^, 40 cycles], 60 °C 1 min [ramp rate 2 °C·s^–1^, 40 cycles], 98 °C 10 min [1 cycle], and 4 °C hold), the 96‐well PCR plate was loaded on a Bio‐Rad QX200 droplet reader and ddPCR data were analyzed with quantasoft analysis software (v. 1.7.4, Bio‐Rad, Hercules, CA, USA). The results were reported as percentage or fractional abundance of target mutant DNA alleles to total (mutant plus wildtype) DNA alleles. Data from multiple wells were combined for mutant allele fraction (MAF) analysis and a minimum of two droplets positive for the mutant allele was required to call a positive timepoint, as previously recommended [24].

### 2.5 Statistical analyses

Patient characteristics were summarized by frequency counts and percentages for categorical variables and medians and ranges for continuous variables. Response to LY2780301 paclitaxel combination was assessed according to RECIST 1.1 criteria [25]. The 6‐month ORR was defined as at least one partial or complete response measured within the first 6 months after treatment initiation. The 6‐month clinical benefit rate was defined as at least one partial or complete response at 6 months after initiation of treatment or disease stabilization during the first 6 months of treatment. Circulating tumor DNA status at baseline was defined as positive if TF > 0 and negative if TF = 0. Correlation of response to treatment with ctDNA status at baseline (ctDNA positive *vs* no detection), ctDNA status after 7 weeks on treatment (TF decreased to 0 or remained stable to 0 *vs* TF remained >0 or became >0) and PI3K/AKT/PTEN pathway alteration in baseline plasma (PI3KAKT+ *vs* PI3KAKT–) was assessed using Fisher's Exact Test. The same statistical test was used for comparisons with categorical clinicopathological features and the rank‐Wilcoxon's test for continuous features. Progression‐free survival (PFS) was defined as the delay between the measurement of interest variable (C1D1 for baseline TF status and PI3K/AKT/PTEN pathway alteration in baseline plasma; ctDNA status at week 7 [or C3D1]) and disease progression or death from any cause. Patients lost to follow‐up or without any event were censored at the date of last contact. PFS probabilities were estimated using the Kaplan–Meier method and subgroups were compared by the log‐rank test. Multivariable Cox proportional hazards regression was used to evaluate potential prognostic factor significance. Hazard ratios (HRs) with their 95% confidence interval (95% CI) were provided and the null assumption (HR = 1) was assessed using the Wald's test. The statistical analyses were carried out using sas v. 9.4 (SAS Institute, Cary, NC, USA) with a nominal level of statistical significance (two‐tailed) set to 0.05. and r (v. 3.5.1; http://www.cran.r‐project.org/). This study was conducted in compliance with the Reporting recommendations for tumor marker prognostic studies (REMARK) criteria [26], see Table S1. NGS, aCGH, and LC‐WGS sequencing data are available in Tables [Link], [Link], [Link].

### 2.6 Ethics considerations

As required by the French regulation, the study protocol was approved by both an Ethics Committee (Comité de Protection des Personnes Sud Méditerranée I) and the French Agency Health authority (ANSM) and was registered as required (N°EUDRACT 2013‐000585‐12, NCT01980277). Patients were enrolled after signature on a written informed consent. All procedures were done in accordance with the 2008 Helsinki Declaration.

## 3 Results

### 3.1 Circulating tumor DNA detection and correlation with outcome

Patients enrolled in the TAKTIC trial were included in five French cancer centers between January 2014 and June 2017. For patients enrolled in the phase II part, median follow‐up was 11.4 months (95% CI, 7.9–17). Six‐month ORR was 63.9% (90% CI, 48.8–76.8) in the overall phase II population, and 55% (90% CI, 35–73.7) in patients with activation of the PI3K/AKT/S6 pathway. Median PFS was 10.6 months (95% CI, 7.6–17.1) and the 6‐month clinical benefit rate was 80.6% (95% CI, 63.4–91.2). Clinical characteristics are summarized in the related clinical article [11]. Characteristics of the population (44 patients) analyzed in this ancillary study were comparable to that of the clinical cohort (Table 1).

**Table 1:** Clinical and molecular characteristics of patients included in the clinical and ancillary cohorts of the TAKTIC trial. Data are expressed as *N* (%) unless otherwise specified.

	Clinical cohort	Ancillary cohort
Number of patients	48	44
Median age (years, range)	51 [29–75]	52.50 [34–75]
Hormone receptor (HR) status		
Negative	3 (6.4)	3 (6.8)
Positive	44 (91.7)	40 (93.2)
Missing data	1	1
Disease stage at inclusion		
Metastatic	48 (100.0)	44 (100.0)
Metastatic sites		
Visceral	43 (89.6)	40 (90.9)
Nonvisceral	5 (10.4)	4 (9.1)
Number of metastatic sites		
< 3	24 (50.0)	23 (52.3)
≥ 3	24 (50.0)	21 (47.7)
Activation of the PI3K/AKT pathway		
PI3KAKT+	22 (53.7)	18 (48.7)
PIKAKT–	19 (46.3)	19 (51.3)
Missing data	7	7
*PI3KCA* mutation (NGS and/or Sanger)		
Yes	12 (29.3)	11 (29.7)
No	29 (70.7)	26 (70.3)
Missing data	7	7
*AKT1* mutation (NGS and/or Sanger)		
Yes	5 (12.2)	4 (10.5)
No	36 (87.8)	33 (89.5)
Missing data	7	7
Absence of PTEN expression by IHC or *PTEN* loss by array‐CGH		
No	36 (85.7)	33 (86.8)
Yes	6 (14.3)	5 (13.2)
Missing data	6	6

**Fig. 1:**
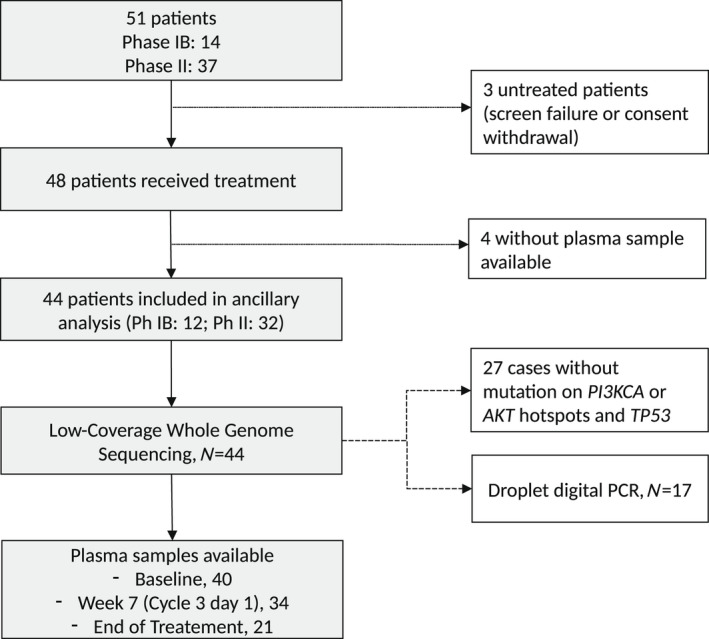
Study flow diagram detailing the patients included in this ancillary analysis and the number of samples evaluated at each timepoint.

Among these 44 patients with at least one plasma sample (including a C1 D1 sample) available with cell‐free DNA of adequate quantity/quality, 12 had been treated in the phase IB part and 32 in the phase II part (Fig. 1). The median cell‐free DNA concentration was 9.75 ng·mL^–1^ at baseline (range 2.2–579.6). It was similar at C3D1 (8.57 ng·mL^–1^; *P* = 0.68, Wilcoxon test baseline *vs* C3D1), but tended to be higher at progression (16.34 ng·mL^–1^; *P* = 0.07, Wilcoxon test C3D1 *vs* progression).

Out of all patients with plasma available, 17 harbored tumor mutations involving *PIK3CA* hotspots, *AKT1* hotspots, or *TP53*. Ten displayed a tumor *PIK3CA* hotspot mutation. Five *PIK3CA* wildtype cases had a *TP53* mutation that could be used for ctDNA monitoring. Two of them also displayed *AKT1* E17K mutations and two others were *PIK3CA* wildtype/*TP53* wildtype/*AKT1* mutated. At least one of these mutations was identified in plasma for all but two patients with cell‐free DNA available at baseline. In cases with *PIK3CA, AKT1*, or *TP53* mutations, plasma MAF assessed by ddPCR and TF from LC‐WGS were correlated (*R* = 0.68, *P* = 0.002). When mutations were detected both in plasma and tumor, plasma MAF (defined by ddPCR) was correlated with tumor MAF assessed by tumor sequencing (*R* = 0.75, *P* = 0.03).

As ddPCR analyses were not available in all cases, and in order to be consistent through the whole set, we considered as ctDNA‐positive all samples with TF > 0 according to LC‐WGS. Twenty‐seven of the 40 patients (67.5%) with baseline plasma samples available had TF > 0. Four more patients with TF = 0 had positive ddPCR assays (Table S5). Clinical and pathological features of patients with positive ctDNA at baseline were similar to those of ctDNA‐negative cases (Table S6).

**Fig. 2:**
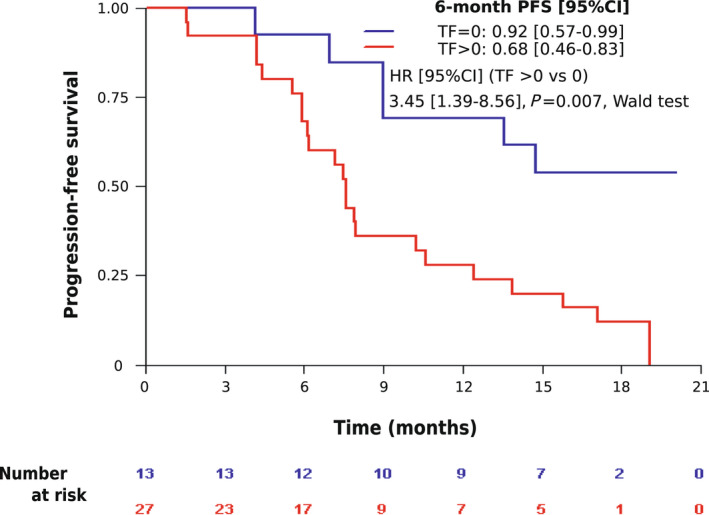
Kaplan–Meier curves for progression‐free survival (PFS) according to baseline circulating tumor fraction (TF). Patients with TF > 0 (red line) display a worse PFS than patients with TF = 0 (blue line). Wald test *P*‐value.

Ct‐DNA status at baseline was not predictive of response to paclitaxel‐LY2780301. Eighteen of 26 (69.2%) ctDNA‐positive patients experienced an objective response under treatment *vs* 7 of 13 (53.8%) ctDNA‐negative cases, (*P* = 0.48). ORR data were missing for one ctDNA‐positive patient. Similar results were observed concerning the clinical benefit rate: 69% *vs* 77% (*P* = 0.72). However, baseline ctDNA detection was associated with PFS. The 6‐month PFS rate was 92% (95% CI, 57–99) for ctDNA‐negative patients *vs* 68% (95% CI, 46–83) for ctDNA‐positive patients (HR = 3.45; 95% CI [1.39–8.56], Fig. 2). Similar results were observed by adding ddPCR results to cases with baseline TF = 0 (data not shown). Multivariate analysis including hormone receptors expression status, number of metastatic sites, the presence of visceral metastasis, and baseline ctDNA status showed that baseline ctDNA status was an independent prognostic marker (Table 2). Adding baseline cfDNA levels to this analysis showed that cfDNA was not prognostic in our cohort without modifying the ctDNA value (data not shown).

**Table 2:** Cox regression multivariate analysis for progression‐free survival.

Variables	Contrast	Hazard ratio [95% CI]	*P*‐value
Hormone receptors expression	Neg *vs* Pos	3.50 [0.90–13.60]	0.07
Number of metastatic sites	≥3 *vs* <3	1.13 [0.50–2.53]	0.77
Visceral metastases	Yes *vs* No	1.63 [0.48–5.53]	0.44
**Baseline TF in plasma**	**>0 *vs* 0**	**3.45 [1.34–8.90]**	**0.01**

Variable significanly associated with progression‐free survival is in bold

**Fig. 3:**
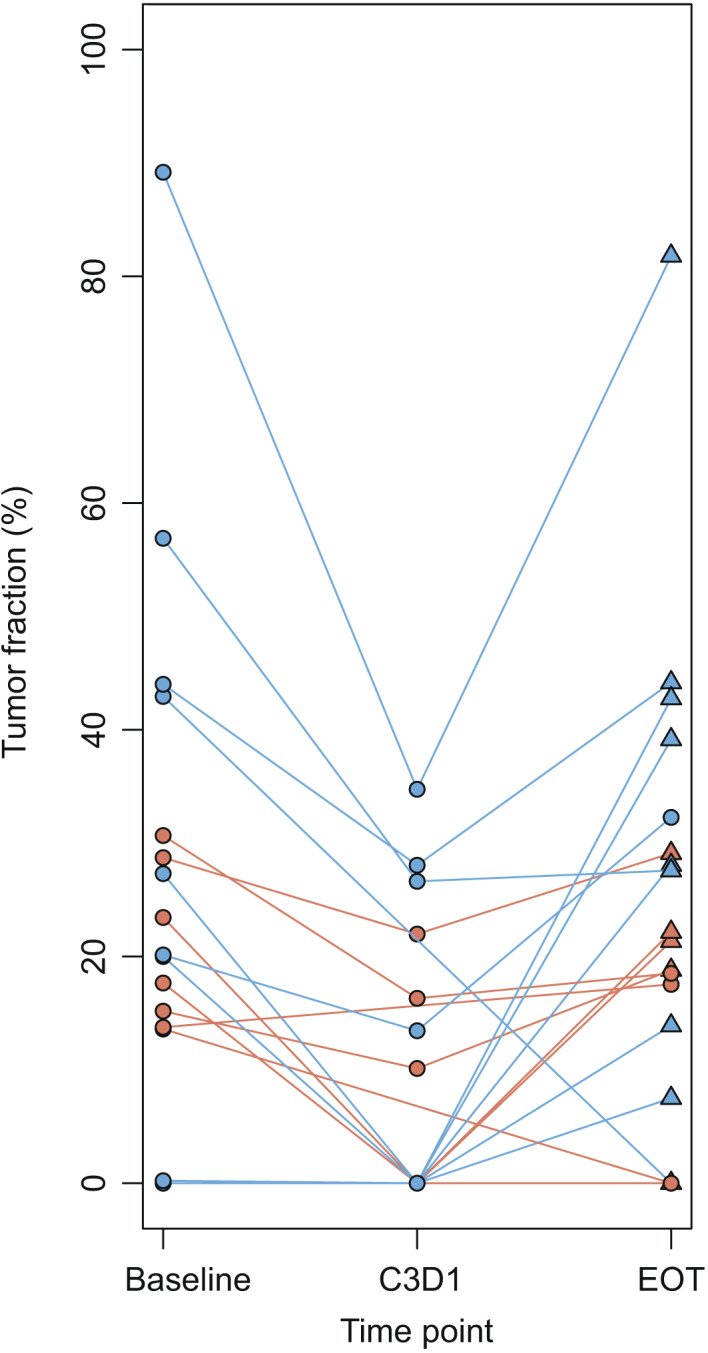
Tumor fraction dynamics under treatment. Data for patients with at least baseline and End of Treatment (EoT) ctDNA samples available are represented (*N* = 19). Blue: responders to paclitaxel‐LY2780301 according to RECIST 1.1 criteria; red: nonresponders to paclitaxel‐LY2780301. Triangles represent cases with tumor progression at time of EoT visit; circles represent cases without tumor progression at time of EoT visit.

Circulating tumor DNA negative status after 7 weeks on treatment was observed for 10 patients: 41.5% of cases with both baseline and C3D1 samples available and ctDNA‐positive status at baseline. CtDNA‐negative status at C3D1 was not associated with response to treatment. Patients who were ctDNA‐negative at C3D1 had an ORR of 55.6% compared to 73.3% for patients who were still or became ctDNA‐positive (*P* = 0.47). This parameter was also not correlated with clinical benefit (*P* = 1) or with PFS (*P* = 0.65, Fig. S1). Limiting ctDNA kinetics assessment (week 7 *vs* baseline) to patients with ctDNA detected at inclusion led to similar results concerning response to treatment: *P* = 1 for 6m‐ORR and 6m‐CBR. CtDNA clearance was not a significant prognostic marker in this small subset (*N* = 23, *P* = 0.23; HR = 1.74 [0.71–4.31]). Additional plasma analysis showed that plasma TF was rising at the time of tumor progression, with a positive predictive value of 86% (Fig. 3).

### 3.2 Qualitative ctDNA assessment by low‐coverage whole‐genome sequencing

#### 3.2.1 Genomic profiles identified by LC‐WGS

**Fig. 4:**
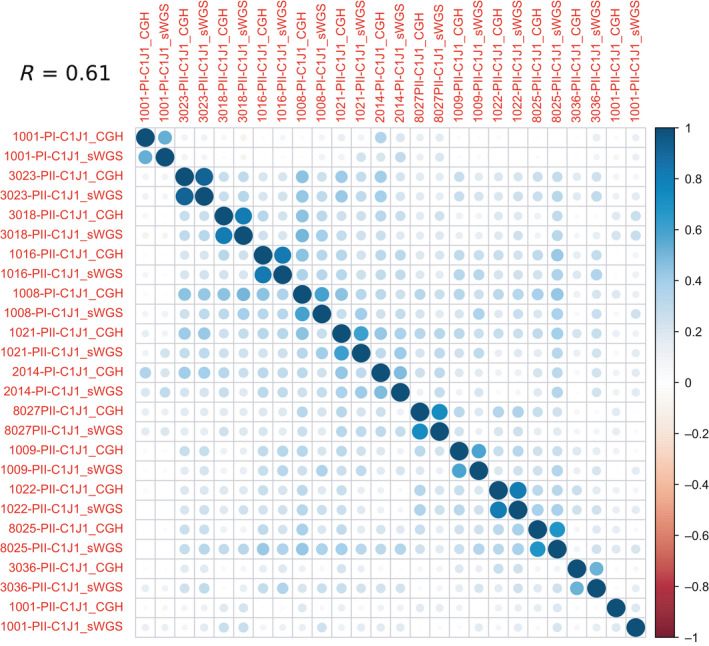
Correlation of baseline tumor and plasma CNA profiles in patients with tumor fraction >0. Analysis was performed using discrete values. Each row/line represents a baseline tumor sample (analyzed with aCGH) or a baseline plasma sample (analyzed with LC‐WGS). Positive correlations are displayed in blue and negative correlations in red, with 1 (dark blue) as the highest correlation. Circles size and color intensity are proportional to the correlation coefficient.

Analysis of copy number alteration profiles of baseline LC‐WGS samples identified several regions with gain/amplification or loss/deletion (Fig. S2). We observed amplification of the 10q11 region including *MAPK8* and the 8q11‐q24 region including *MYC, CCNE2, CCN3*, and *E2F5*, all involved in cell cycle and proliferation. Regions of chromosomes 1q (*VANGL2*), 17q (*MAP2K6, BIRC5, CCL2, PRKCA, BRIP1*), and 7p (*HOXA1, HOXA5*) were also frequently amplified (Table S7). Major tumor suppressor gene losses were also observed, including *FOXP1* (3p13), *RB1* (13q14), and *TP53* (17p13). Plasma genomic profiles were close to their tumor counterparts. CNA profiles of cases with positive TF at baseline clustered with the corresponding tumors (Fig. 4). The correlation coefficient (*R* = 0.61) suggests a correlation between plasma and tumor CNA profiles, but should be interpreted with caution due to the limited sample size of this subset.

#### 3.2.2 PI3K/AKT/mTOR pathway alterations can be assessed by LC‐WGS

As the TAKTIC trial explored the efficacy of an AKT/S6‐inhibitor, we focused LC‐WGS analyses on genes involved in the PI3K/AKT/mTOR pathway. Nine patients had *PTEN* loss in plasma at baseline. Of them, we retrieved all five patients classified as PTEN‐negative in tumor (by IHC or aCGH). Two were classified as PTEN‐positive in tumor tissue, suggesting that MBC from these patients may be heterogeneous and/or *PTEN* loss could be subclonal. Baseline tumor status was not available for two cases.

Thirty‐one of 40 (77.5%) cases with baseline plasma sample available displayed circulating copy number alterations of genes involved in the PI3K/AKT/mTOR pathway. Regions altered in at least 15 cases involved *PIK3R2* and *STK11* (19p13), *PIK3C2B* (1q32), *AKT3* (1q44), *TSC1* (9q34), *RPS6KB1* (17q23), and *AKT1* (14q32). Fifteen cases displayed *AKT1* gain/amplification in plasma, 22 *STK11* loss, 9 *PIK3CA* gain, and 7 *mTOR* or *RPS6KB1* gain. Of note, there was a good correlation between plasma and tumor genomic profiles when focusing on genes involved in the PI3K/AKT/mTOR pathway (Fig. S3). The highest correlations were found for *PIK3CA* (100% accuracy)*, PIK3R1* (85% accuracy)*, PTEN, FBXW7, PIK3C2B, and PIK3CB* (77% accuracy); see Fig. S4. A majority of genes classified as discordant between tumor and plasma had circulating CNAs not observed in tumor (57% *vs* 43%).

Detection of at least one PI3K/AKT/PTEN pathway alteration in baseline plasma sample was not correlated with response to treatment when assessed alone (*P* = 0.70) or in combination with tumor status (*PIK3CA* or *AKT1* mutations or *PTEN* deletion, *P* = 1). PFS was also not significantly associated with plasma PI3K/AKT/PTEN pathway status (HR = 0.48, 95% CI [0.18–1.27]).

### 3.3 LC‐WGS can identify CNAs emerging at the time of tumor progression

To explore CNAs potentially associated with secondary resistance to paclitaxel‐LY2780301 treatment, we compared the CNA profiles of plasma samples at inclusion and at progression for patients with both samples available (*N* = 15) using the GISTIC tool. Main alterations identified at inclusion were also detected at progression. Seventy‐five percent of CNAs identified at baseline were still observed at progression (Fig. S5). Losses were significantly more frequently conserved than gains (median = 83.3% *vs* 71.4%, *P* = 0.0077, Wilcoxon test).

Among the alterations more common in post‐progression samples, we identified losses of 3p21 and 3p13 regions including *SETD2* and *FOXP1* and gains of the 1q41 (*TGFB2*), 1q42 (*MAP3K21/MLK4*), 2q11 (*MAP4K4*), 10q11 (*RET*), and 10q22 (*CDH23*) regions (Table S8).

## 4 Discussion

AKT inhibitors are promising drugs for MBC treatment with several ongoing or recently completed clinical trials but no approval to date. Plasma low‐coverage whole‐genome sequencing of plasma samples from the TAKTIC phase I/II trial shows that ctDNA quantitative assessment is not predictive of response rate with LY2780301/paclitaxel, but was predictive of PFS.

Even though the LOTUS trial showed a clinical benefit of AKT inhibition in patients with TNBC [13, 27], a recently published randomized phase II trial failed to show a benefit from another AKT inhibitor for ER‐positive cases [8]. This lack of efficacy may be due to our inability to identify specific predictive features. In the BEECH trial, the capivasertib/paclitaxel combination was not more efficient in *PIK3CA*‐mutated tumors than in the whole population. Two hypotheses may be explored to improve our ability to identify the right candidates for these therapies. The first is to extend the molecular assessment to other alterations. For example, we can add other PI3K/AKT/mTOR pathway alterations such as *AKT* mutation, *PTEN* deletion, or functional assays. However, analysis of PI3KCA/AKT1/PTEN tumor alterations in patients included in the TAKTIC trial did not show any difference concerning the response to paclitaxel‐LY2780301 [11]. Another way may be to improve the sensitivity to be sure to detect all positive cases for a specific alteration. Genotype modification through disease evolution and disease heterogeneity may indeed limit the predictive value of single‐tissue biopsies. Comprehensive assessment of tumor‐associated alterations may be reached by using ctDNA [28, 29, 30, 31]. Unfortunately, quantitative assessment of ctDNA levels (tumor fraction) by LC‐WGS in the present cohort did not achieve this goal.

Nevertheless, exploring specific alterations of genes involved in the biological pathways of interest may overcome this limitation. Regarding the PI3K/AKT pathway, despite only 49% of the patients included in the TAKTIC trial harboring PI3K/AKT alterations in tumor, this rate rose to 78% when plasma alterations were taken into account. This further emphasizes that MBC is heterogeneous and that single‐core biopsies are not sufficient to identify all subclonal molecular alterations, as has been described in breast cancer and other tumor types [32, 33, 34, 35].

The ability of circulating alterations to predict outcome has already been explored concerning PI3K and AKT inhibitors. In the BELLE‐2 and SOLAR‐1 trials, buparlisib and alpelisib (both PI3K inhibitors) efficacy was correlated with detection of circulating *PI3KCA* mutations [21, 36]. Whereas capivasertib‐paclitaxel combination failed to improve PFS in ER+/HER2‐ MBC, early ctDNA decrease under treatment was associated with survival [37]. However, this prognostic value was also observed in patients receiving paclitaxel/placebo, and was thus not specific of capivasertib efficacy. Ipatasertib is another AKT inhibitor known to improve survival in triple‐negative MBC [13]. In that trial, baseline ctDNA was correlated with prognosis (PFS) in both treatment arms [38]. This is therefore consistent with our study showing that ctDNA is correlated with survival, but no clear evidence exists to confirm that it can specifically predict AKT inhibitors efficacy. From a quantitative point of view, we observed that ctDNA levels were concomitant with disease progression in most patients included in the TAKTIC trial. This suggests that ctDNA assessment may be used as a surrogate of tumor progression and be complementary to radiological evaluations in MBC [39], as reported in the early setting to anticipate the disease recurrence [40, 41]. In some hormone‐receptors‐positive MBC, *ESR1* mutation detection antedates tumor progression and may be a useful biomarker to adapt endocrine therapy [42, 43, 44].

Beyond prognosis, we identified CNAs associated with disease progression under paclitaxel‐LY2780301. *SETD2* is frequently mutated or deleted in various cancers [45]. Deletions have been reported in clear‐cell renal cancer [46], colorectal cancer [47], leukemia [48], sarcoma [49], and lung adenocarcinoma [50]. Alterations of the histone methyltransferase SETD2 are also correlated with prognosis in renal cell cancer and may be involved in resistance to chemotherapy in breast cancer, as was observed with paclitaxel in our study [51, 52]. *FOXP1* losses are more common in progressive samples in our set. *FOXP1* deletion is involved in prostate cancer and neuroblastoma proliferation and is associated with prognosis [53, 54]. AKT/mTOR inhibition in breast cancer cell lines leads to decreased *FOXP1* expression [55]. Treatment with LY2780301 may have induced *FOXP1* loss in the present study. The *TGFB2* copy number is higher in post‐progression samples. TGFB signaling is involved in epithelial‐mesenchymal transition (EMT), cell motility, tumor angiogenesis, and metastasis. In TNBC, high *TGFB2* expression is associated with poor prognosis and TGFB inhibition in BC cell lines may decrease tumor invasion [56, 57]. This suggests that addition of TFGB inhibitors should be explored to overcome resistance to LY2780301/paclitaxel. Mitogen‐activated protein kinases (MAPK), here MAP3K2 and MAP4K4/MLK4, are also involved in EMT and promote cell proliferation and invasion, as well as resistance to endocrine therapy. Moreover, MAPK interaction with the PI3K/AKT pathway leads to chemoresistance [58]. A combination of PI3K/AKT and MAPK inhibitors may by promising to reverse treatment failure [59]. For example, *MLK4* expression is correlated with tumor invasion and migration [60]. The higher *MLK4* copy number observed in post‐progression plasma samples in our study may be involved in resistance to the LY2780301/paclitaxel combination.

This work has limitations. First, the fact that the TAKTIC trial was a single‐arm study limits its capacity to differentiate the prognostic value of ctDNA to its ability to predict treatment efficacy. Controlled studies involving a comparative arm with paclitaxel alone would be of interest. However, its multicenter prospective design strengthens the value of the observations we made. Of note, LC‐WGS did not predict ORR, suggesting that circulating genomic alterations may be more related to the development of therapeutic resistance than with initial treatment sensitivity. Second, the medium sample size (44 patients with plasma samples available) and the heterogeneity regarding the number of plasma samples available at each timepoint could have reduced the statistical validity of our results. Early ctDNA clearance after treatment initiation has been described to be associated with outcome [37, 38, 61]. We did not observe a correlation between ctDNA negative status after 7 weeks under treatment and response to treatment. Some weaknesses of this work may explain this discrepancy. First, LC‐WGS sensitivity decreases for tumor fraction below 3%. Combination of LC‐WGS to more sensitive technologies, such as ddPCR, could enhance ctDNA detection rates. However, ddPCR requires previous identification of molecular alterations on tumor biopsies. Extending ddPCR to cases with *PI3KCA* and *AKT* hotspot mutations or *TP53* mutations did not improve sensitivity in our set. Second, our population was heterogeneous, with some ER‐negative tumors (7%) and with a mix of patients receiving paclitaxel‐AKTi combination as the first‐line treatment (phase II part) and patients who received prior treatment for their metastatic disease (phase I part). We chose to combine both subsets due to the small sample size and because we did not observe any imbalance between cohorts according to other clinical and pathological features. Third, simultaneous tracking of several alterations (such as cell‐free DNA methylation profiles) would have been of interest to improve the ctDNA clinical value [34, 62, 63]. Development of technologies not dependent on *a priori* knowledge of previously identified alterations can indeed help to explore alterations linked to tumor heterogeneity. The use of LC‐WGS was a choice to explore one aspect of this heterogeneity with a nonexpensive technology requiring a limited amount of cell‐free DNA, as less than 20 ng of total cell‐free DNA was isolated in half of the baseline sample [64]. However, using a higher amount of cell‐free DNA may have increased LC‐WGS sensitivity. Finally, adding additional timepoints during treatment period would have allowed exploring the ctDNA ability to antedate tumor progression and to propose an early change of therapeutic to “ctDNA‐progressive” patients. Nevertheless, additional sample collection was not preplanned in the original clinical study.

## 5 Conclusions

CtDNA status at treatment initiation was correlated with PFS under LY2780301/paclitaxel in the TAKTIC trial. Low‐coverage whole‐genome sequencing identified putative genomic alterations involved in resistance to treatment, suggesting potential therapeutic targets that might be ultimately used to tailor subsequent therapies.

## Conflict of interest

A.G. declares nonfinancial support from Novartis. R.S. declares research grants from EISAI and AstraZeneca; advisory board for Roche, GSK, and Novartis; nonfinancial support (travel, accommodation and meeting registration fees) was from Pfizer, Roche, GSK, BMS, and AstraZeneca. The other authors declare no conflicts of interest.

## Author contributions

RS, CV, and AG designed the study. SG, NC, and JA performed ctDNA experiments. ArG performed all bioinformatics analyses. RS, NI, FD, MC, CL, CV, FB, and AG enrolled patients and collected clinical data. JP was responsible for clinical trial management. PS and ArG performed all statistical analyses. All authors contributed to data interpretation, article writing, and approval of the submitted version.

### Peer review

The peer review history for this article is available at https://publons.com/publon/10.1002/1878‐0261.13188.

## Supporting information


**Fig. S1.** Kaplan–Meier curves for progression‐free survival (PFS) according to circulating tumor fraction (TF) at week 7 (C3D1).
**Fig. S2.** Frequency plot of copy number alterations identified in baseline plasma samples. Frequencies of gains and losses are plotted as a function of chromosome location. Vertical lines represent chromosome boundaries. Positive and negative values indicate frequencies (Log‐scale) of tumors showing copy number increase (red) and decrease (green).
**Fig. S3.** Correlation of tumor and baseline plasma CNA involving genes of the PI3K/mTOR/AKT pathway in patients with tumor fraction > 0. Analysis was performed using discrete values. Each row/line represents a baseline tumor sample (analyzed with aCGH) or a baseline plasma sample (analyzed with LC‐WGS). Positive correlations are displayed in blue and negative correlations in red, with 1 (dark blue) as the highest correlation. Circles size and color intensity are proportional to the correlation coefficient.
**Fig. S4.** Comparison of copy number alterations (tumor *vs* plasma) in genes of the PI3K/AKT pathway in patients with both tumor and plasma samples available at baseline and with tumor fraction > 0 at baseline. Each bar represents one gene and is dichotomized between cases with both tumor and plasma samples altered (blue) and cases with discrepant gene status (red).
**Fig. S5.** Genomic identification of significant targets in cancer (GISTIC) copy number alterations profiles in plasma at baseline (top) and progression (bottom). Frequencies of gains and losses are plotted as a function of chromosome location. Vertical lines represent chromosome boundaries. Positive and negative values indicate GISTIC score of tumors showing copy number increase (red) and decrease (green). Blue dotted lines represent thresholds for significance (*P* < 0.05).

**Table S1.** REMARK checklist.

**Table S2.** Tumor NGS data.

**Table S3.** Tumor aCGH data.

**Table S4.** Plasma low‐coverage – WGS data.

**Table S5.** Details of plasma samples available and ctDNA assessment results.

**Table S6.** Demographics according to baseline ctDNA status.

**Table S7.** GISTIC analysis of baseline plasma copy number alteration profiles.

**Table S8.** GISTIC comparison of baseline and progression plasma copy number alteration profiles.

## Data Availability

All data generated or analyzed during this study are included in this published article (Supplementary data files).
