# Supplementary material for: Circulating tumor DNA predicts efficacy of a dual AKT/p70S6K inhibitor (LY2780301) plus paclitaxel in metastatic breast cancer: plasma analysis of the TAKTIC phase IB/II study
Source: Mol Oncol. 2022 Mar 30;16(10):2057–70. doi: 10.1002/1878-0261.13188 (PMC9120890; doi:10.1002/1878-0261.13188)

Supplementary Figure 1

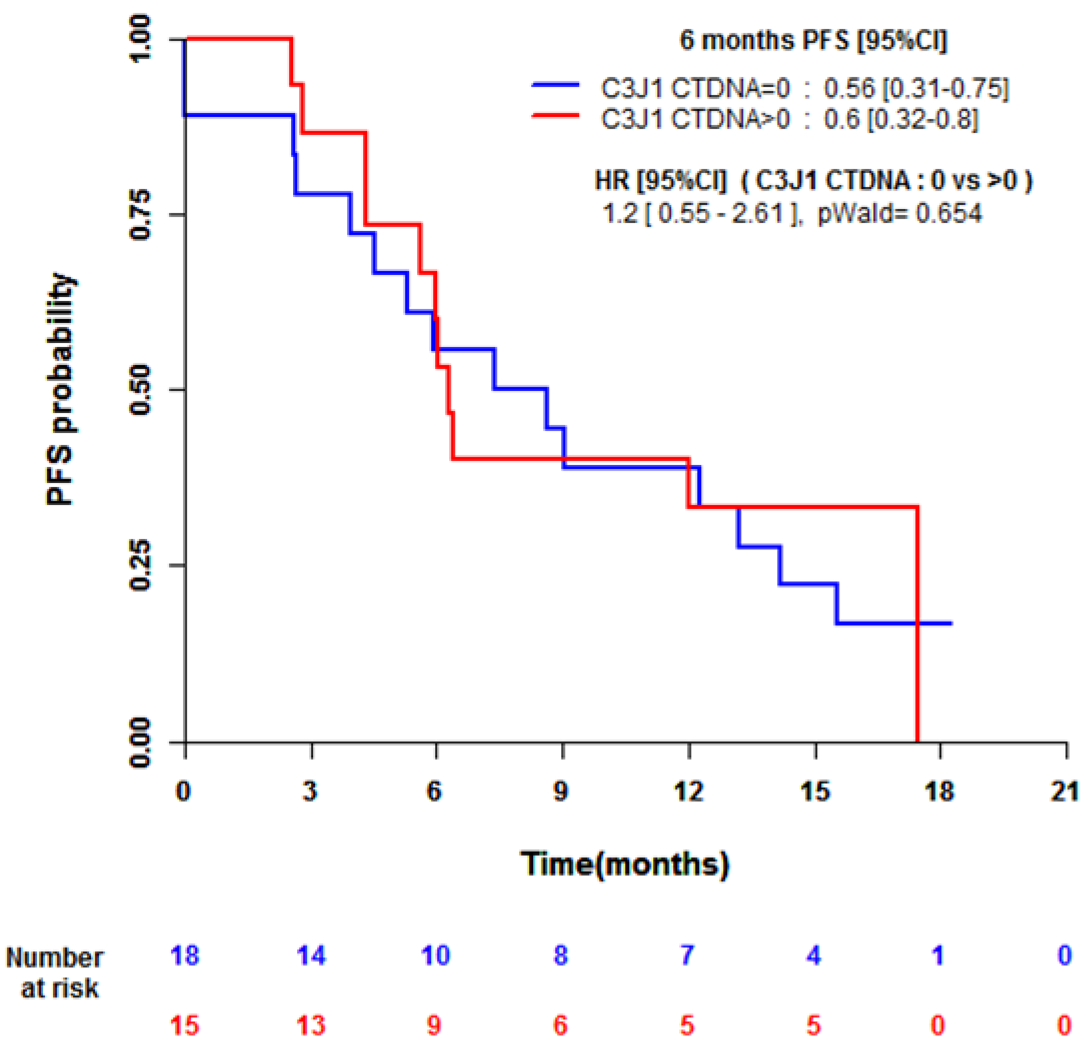

Supplementary Figure 2

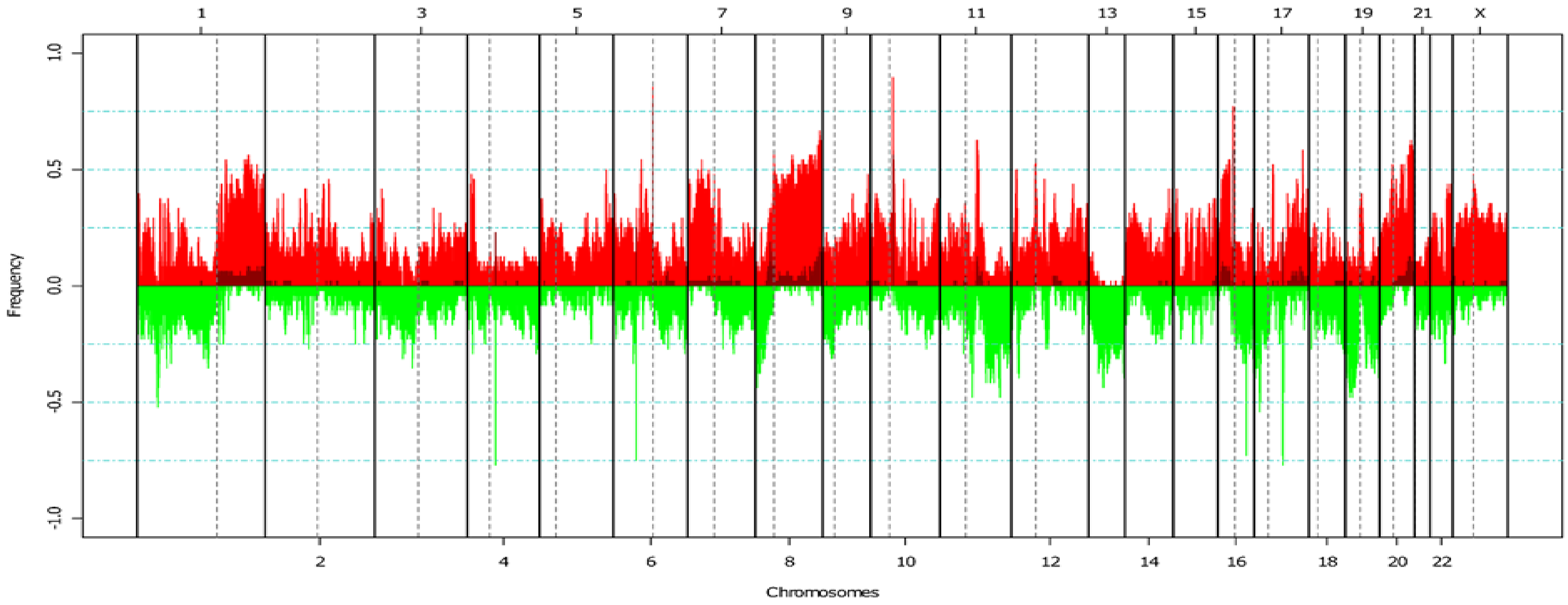

Supplementary Figure 3

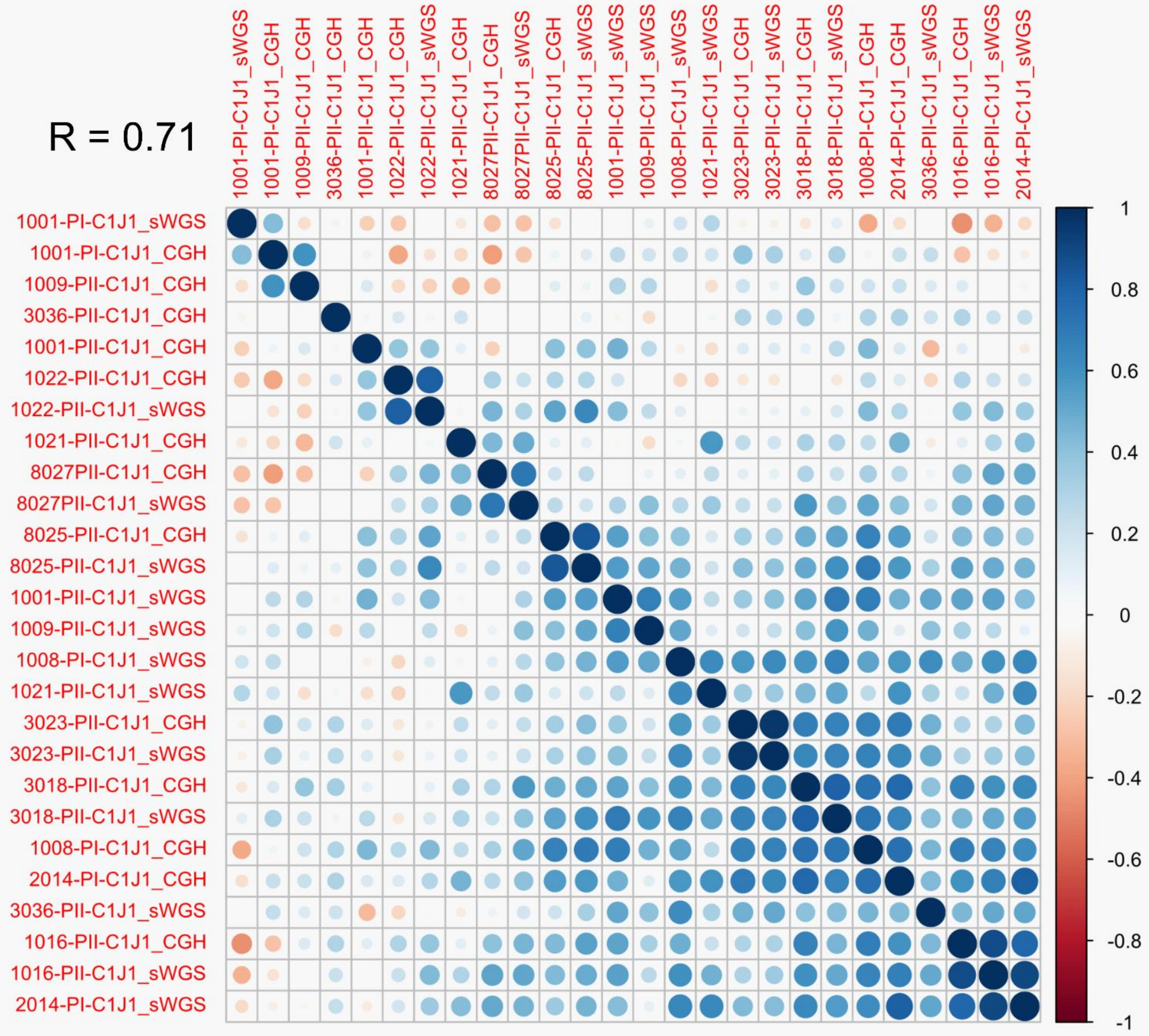

Supplementary Figure 4

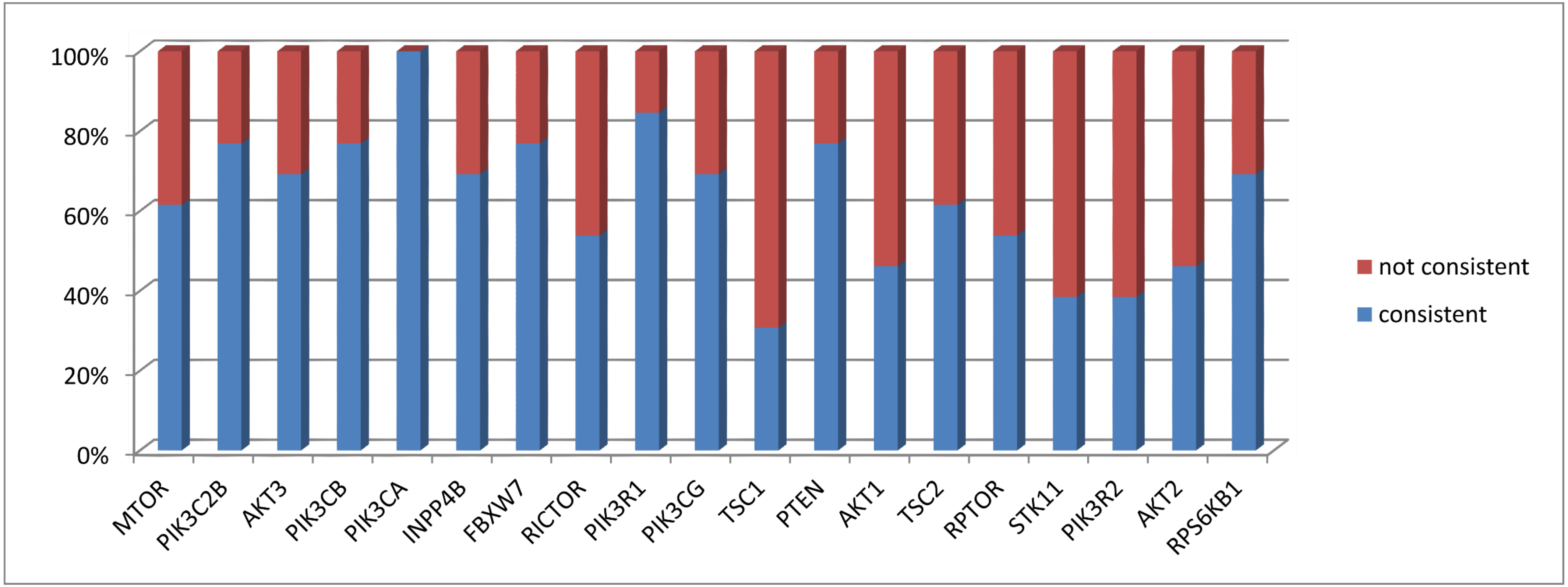

Supplementary Figure 5

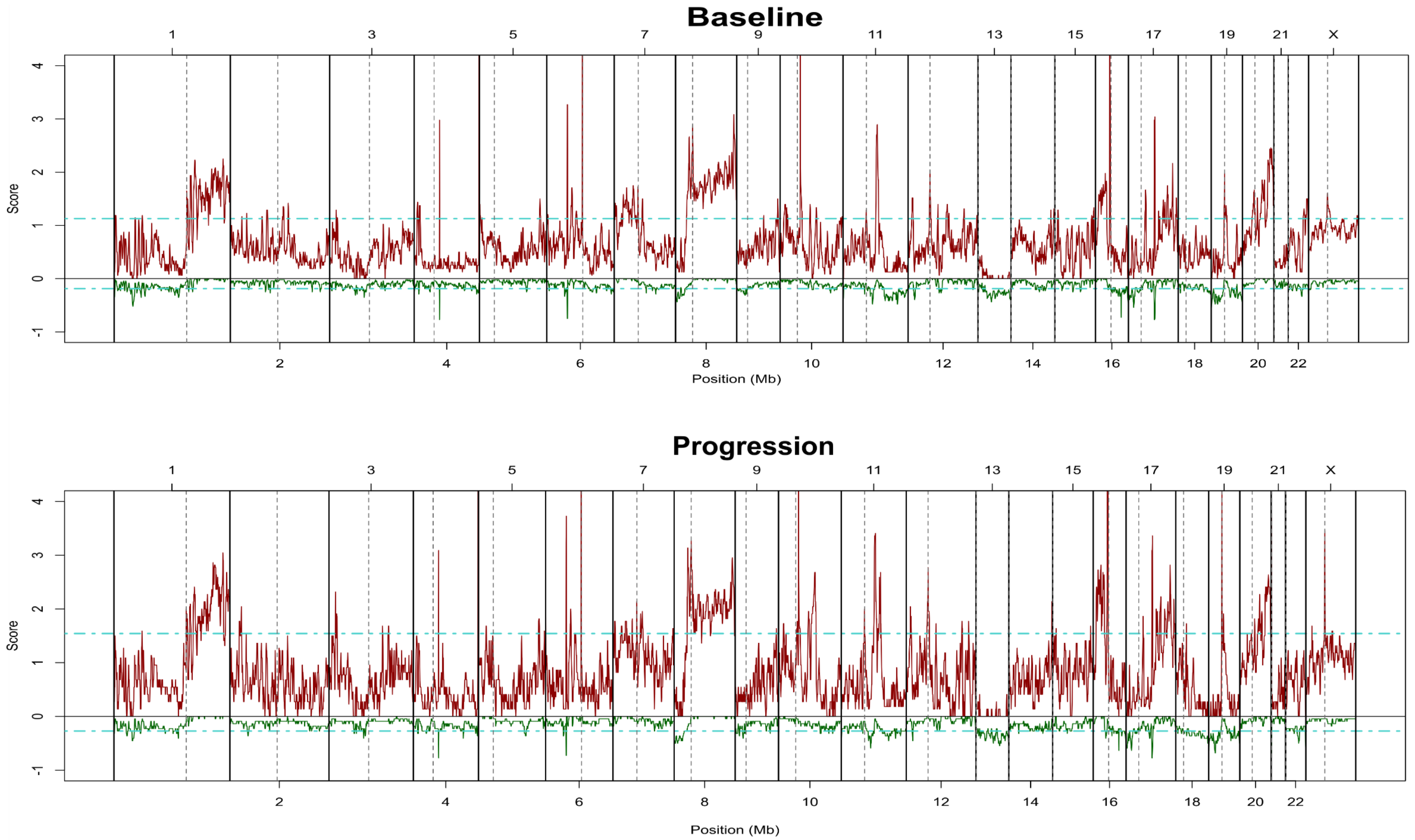

Supplement: Supplementary file 1 — Fig. S1. Kaplan–Meier curves for progression‐free survival (PFS) according to circulating tumor fraction (TF) at week 7 (C3D1). Fig. S2. Frequency plot of copy number alterations identified in baseline plasma samples. Frequencies of gains and losses are plotted as a function of chromosome location. Vertical lines represent chromosome boundaries. Positive and negative values indicate frequencies (Log‐scale) of tumors showing copy number increase (red) and decrease (green). Fig. S3. Correlation of tumor and baseline plasma CNA involving genes of the PI3K/mTOR/AKT pathway in patients with tumor fraction > 0. Analysis was performed using discrete values. Each row/line represents a baseline tumor sample (analyzed with aCGH) or a baseline plasma sample (analyzed with LC‐WGS). Positive correlations are displayed in blue and negative correlations in red, with 1 (dark blue) as the highest correlation. Circles size and color intensity are proportional to the correlation coefficient. Fig. S4. Comparison of copy number alterations (tumor vs plasma) in genes of the PI3K/AKT pathway in patients with both tumor and plasma samples available at baseline and with tumor fraction > 0 at baseline. Each bar represents one gene and is dichotomized between cases with both tumor and plasma samples altered (blue) and cases with discrepant gene status (red). Fig. S5. Genomic identification of significant targets in cancer (GISTIC) copy number alterations profiles in plasma at baseline (top) and progression (bottom). Frequencies of gains and losses are plotted as a function of chromosome location. Vertical lines represent chromosome boundaries. Positive and negative values indicate GISTIC score of tumors showing copy number increase (red) and decrease (green). Blue dotted lines represent thresholds for significance (P < 0.05). [file MOL2-16-2057-s002.pdf]
